# JKO schemes with general transport costs

**DOI:** 10.1007/s00526-026-03256-x

**Published:** 2026-02-17

**Authors:** Cale Rankin, Ting-Kam Leonard Wong

**Affiliations:** 1 Department of Mathematics, Monash University, Victoria, Australia; 2 Department of Statistical Sciences, University of Toronto, Toronto, Canada

**Keywords:** 35K57 (Primary), 58J35, 82C31 (Secondary)

## Abstract

We modify the JKO scheme, which is a time discretization of the Wasserstein gradient flow, by replacing the Wasserstein distance with more general transport costs on manifolds. We show when the cost function has a mixed Hessian which defines a Riemannian metric, our modified JKO scheme converges, under suitable conditions, to the corresponding Riemannian Fokker–Planck equation. Thus on a Riemannian manifold one may replace the (squared) Riemannian distance with any cost function which induces the metric. Of interest is when the Riemannian distance is computationally intractable, but a suitable cost has a simple analytic expression. We consider the Fokker–Planck equation on compact submanifolds with the Neumann boundary condition and on complete Riemannian manifolds with a finite drift condition. As an application we consider Hessian manifolds, taking as a cost the Bregman divergence.

## Introduction

In this paper we modify the JKO scheme, which was named after the seminal work of Jordan, Kinderlehrer and Otto [1], so as to encompass optimal transport costs other than the Wasserstein distance. We begin by reviewing the JKO scheme and explaining why one would want to modify it.

The JKO scheme is a physically meaningful, iterative scheme for approximating the solutions to dissipative partial differential equations which are gradient flows in the Wasserstein space. Taking for granted some standard terminology (see Table 1 and Section 2), the scheme for the heat equation is as follows: Let (*M*, *g*) be a Riemannian manifold, $\mathcal {W}_2$ the (quadratic) Wasserstein distance on $\mathcal {P}(M)$, and $\rho _0$ a probability density with respect to the Riemannian volume measure. Fix $\tau >0$, set $\rho ^\tau _0 = \rho _0$ and recursively define

JKO $$\begin{aligned} \rho ^\tau _{k+1} := \text {argmin}_{\rho \in \mathcal {P}(M)} \int _{M} \log \rho \, \textrm{d}\rho + \frac{1}{2\tau }\mathcal {W}^2_2(\rho ,\rho ^\tau _{k}), \end{aligned}$$

where $\int _M \log \rho \textrm{d}\rho$ is the entropy functional. Provided minimizers exist we may define the piecewise constant interpolation

1 $$\begin{aligned} \rho ^\tau (t) := \rho ^\tau _k \quad \text {for } t\in ((k-1)\tau ,k\tau ], \end{aligned}$$

with $\rho ^\tau (0) =\rho _0$. Jordan, Kinderlehrer, and Otto [1], building upon De Giorgi’s minmizing movements scheme [2], proposed the update rule (JKO) in the case $M = \textbf{R}^n$ and showed that as $\tau \rightarrow 0$ the curves $\rho ^\tau$ converge weakly to the solution of the heat equation (they studied more generally the Fokker–Planck equation which amounts to adding a drift term (see (7)) in (JKO)). Their result spurred a tremendous amount of related work, including gradient flows on metric spaces, probabilistic interpretations, as well as numerical implementations and applications. For further details see [3–6] and the references therein.

The Wasserstein distance appearing in (JKO) is defined by

$$\begin{aligned} \mathcal {W}_2^2(\rho ,\rho ^\tau _{k}) = \inf _{\pi \in \Pi (\rho ,\rho ^\tau _k)} \int _{M \times M} d^2(x,y) \, \textrm{d}\pi (x,y), \end{aligned}$$

where $\Pi (\rho ,\rho ^\tau _k)$ is the set of Borel probability measures on $M \times M$ with marginals $\rho$ and $\rho ^\tau _k$, and *d* is the Riemannian distance on (*M*, *g*). The Wasserstein distance is a natural distance on the space of probability measures [7, 8]. However, for many manifolds one does not have simple explicit formulae for the Riemannian distance. In this paper we show if *c*(*x*, *y*) is any (possibly asymmetric) $C^3$ cost function on $M \times M$ such that in coordinates

2 $$\begin{aligned} g_{ij}(x) = -c_{x^i,y^j}(x,x), \end{aligned}$$

then one may replace the term $\frac{1}{2}\mathcal {W}_2^2(\rho , \rho _k^{\tau })$ in (JKO) by the transport cost

$$\begin{aligned} \mathcal {T}_{c}(\rho ,\rho ^\tau _{k}) = \inf _{\pi \in \Pi (\rho ,\rho ^\tau _k)} \int _{M \times M} c(x,y) \, \textrm{d}\pi (x,y), \end{aligned}$$

and obtain a sequence of interpolants which converge to the same limit as the original scheme. An alternate viewpoint of this result is when (2) defines a Riemannian metric, the JKO scheme with $\mathcal {T}_{c}$ in place of $\frac{1}{2}\mathcal {W}_2^2$ yields approximate solutions to the Riemannian heat or Fokker–Planck equation. While the limiting PDE is the same, the advantage occurs when, for a given metric *g*, one may find a cost *c* realizing (2) for which *c*(*x*, *y*) is significantly easier to compute than *d*(*x*, *y*).

Such a case motivates our modification. As an application of our main result, stated in Theorem 1 below, we consider Hessian manifolds with a global chart, equivalently $M \subset \Omega$ where $\Omega$ is an open convex domain in $\textbf{R}^n$ equipped with a Riemannian metric of the form $g_{ij}(x) = \varphi _{ij}(x):= \varphi _{x^ix^j}(x)$ for $\varphi$ a convex function. Letting $\varphi (x) = \frac{1}{2}|x|^2$ recovers the Euclidean metric. Hessian Riemannian metrics have been intensively studied in differential geometry [9, 10] and optimization theory [11, 12], yet there is no simple formula for the Riemannian distance except in special cases. However, the Bregman divergence

3 $$\begin{aligned} c(x,y) = B_{\varphi }(x,y) := \varphi (x)-\varphi (y)-D\varphi (y)\cdot (x-y), \end{aligned}$$

satisfies (2) and is straightforward to compute. Thus on such a Hessian manifold one may compute approximate solutions to the heat equation and Fokker–Planck equation using a JKO scheme with transport cost

4 $$\begin{aligned} \mathcal {B}_{\varphi }(\rho ,\rho ^\tau _{k}) := \inf _{\pi \in \Pi (\rho ,\rho ^\tau _k)} \int _{\Omega \times \Omega } B_{\varphi }(x,y) \, \textrm{d}\pi (x,y). \end{aligned}$$

The transport cost (4) was first considered by Carlier and Jimenez [13] and, more recently, the authors [14] studied the geometric structures on $\mathcal {P}(M)$ induced by (4), which we call the Bregman–Wasserstein divergence. We note that computation of (4) is, up to a coordinate transformation (see [14, Proposition 3.2] and (53) below), equivalent to that of a Euclidean Wasserstein distance for which Brenier’s theorem applies and many computational algorithms are now available. Since the Bregman divergence has numerous applications in statistics and applied mathematics (see for example [15–18]), we believe the corresponding modified JKO scheme will be useful for studying related problems involving optimal transport. Regarding the use of general cost functions, we also note the recent work of Léger and Aubin-Frankowski [19] who developed a unifying perspective of gradient descent algorithms using generalized convex duality from optimal transport.

Before we provide additional background references, we state our main theorems. Our terminology and precise statements of our assumptions are provided in Section 2. We will prove our main result for arbitrary costs on Riemannian manifolds. Then as a corollary we list our motivating case: the Bregman divergence on a subset of $\textbf{R}^n$. Let (*N*, *g*) be an ambient Riemannian manifold with metric *g*, distance *d*(*x*, *y*) and volume measure $\textrm{dVol}_{g}$, and let $M \subset N$ be an open submanifold. Assume $c:N \times N \rightarrow \textbf{R}_+$ is a cost function satisfying (2). Let $\rho _0$ be a probability density on *M* (with respect to the Riemannian volume) which has finite entropy. Our modified JKO scheme for the Riemannian Fokker–Planck equation

$$\begin{aligned} \partial _t\rho = \beta ^{-1} \Delta \rho + \textrm{div}(\rho \nabla \psi ), \end{aligned}$$

where $\beta > 0$ is a constant and $\psi : M \rightarrow \textbf{R}_+$ is a smooth bounded potential satisfying $\Vert \nabla \psi \Vert \le C(1+\psi )$, is the recursion

5 $$\begin{aligned} \rho ^\tau _{k+1} := \text {argmin}_{\rho \in \mathcal {P}(M)} \, \beta ^{-1} \int _{M} \log \rho \, \textrm{d}\rho + \int _{M} \psi \, \textrm{d}\rho + \frac{1}{\tau }\mathcal {T}_{c}(\rho ,\rho ^\tau _{k}). \end{aligned}$$

It will be shown that $\rho ^\tau _{k+1}$ is well-defined as the unique minimizer. Let $\rho ^\tau (t)$, $t \ge 0$, be the piecewise constant interpolants defined by (1).

### Theorem 1

Assume *c* is a $C^3$ cost function on *N* satisfying (2) as well as conditions A1, and A2. Assume further *N* is *c*-convex with respect to itself and that there are constants $\lambda ,\Lambda > 0$ such that $\lambda d^2(x,y) \le c(x,y) \le \Lambda d^2(x,y)$ for $x, y \in M$. Assume either:

(i) *M* is pre-compact in *N* with (non-empty) smooth boundary; or
(ii) $M=N$ and (*M*, *g*) is a complete Riemannian manifold with Ricci curvature bounded below.

Then there exists a measurable function $\rho : [0,\infty ) \times M \rightarrow \textbf{R}_+$ such that for each $t \in [0,\infty )$

$$\begin{aligned} \rho ^\tau (t) \rightharpoonup \rho (t) \text { weakly in }L^1(M) := L^1(M; \textrm{dVol}_{g}), \end{aligned}$$

and $\rho$ solves the Fokker–Planck equation

$$\begin{aligned} {\left\{ \begin{array}{ll} \partial _t\rho = \beta ^{-1} \Delta \rho + \textrm{div}(\rho \nabla \psi ) & \text { in}\quad (0,\infty ) \times M;\\ \rho (0) = \rho _0 & \text { on}\quad M. \end{array}\right. } \end{aligned}$$

Moreover, in case (i) $\rho$ is the unique classical solution satisfying the Neumann boundary condition, and in case (ii) $\rho$ is the unique classical solution with finite drift (by which we mean $\int _M \psi \textrm{d}\rho (t) < \infty$) and second moment.

In the above $\Delta$ and $\textrm{div}$ are the Laplace-Beltrami operator and Riemannian divergence. Table 1 outlines our notation. We list as a corollary the case of most interest to us; further details, and examples, of the Bregman case are given in Section 6.

### Theorem 2

Assume $N=\Omega$ is an open convex subset of $\textbf{R}^n$. Assume $c(x,y) = B_{\varphi }(x,y)$ is a Bregman divergence, where $\varphi : \Omega \rightarrow \textbf{R}$ is smooth convex function such that $D^2 \varphi$ is positive definite on $\Omega$ and $D\varphi (\Omega )$ is convex. Assume either:

(i) *M* is an open and precompact subset of *N* with smooth boundary; or
(ii) $M = N$ and (*M*, *g*), where $g_{ij}(x) = D_{ij}\varphi (x)$ under the Euclidean coordinates on $\Omega$, is a complete Riemannian manifold with Ricci curvature bounded below and there are constants $\lambda ,\Lambda > 0$ such that $\lambda d^2(x,y) \le B_{\varphi }(x,y) \le \Lambda d^2(x,y)$ for $x, y \in \Omega$.

Then the same conclusions as in Theorem 1 hold.

To conclude this introduction we provide a bit more history, and mention other works which have modified the JKO scheme.

Gradient flow schemes in the Wasserstein space were first studied by Otto in conjunction with pattern formation in magnetic fluids [20]. The connection to the Fokker–Planck equation was realized by Jordan, Kinderlehrer, and Otto [1]. Their work indicated there should be a differential structure for which the heat equation is a gradient flow. This structure was found by Otto [21] and was further developed by Ambrosio, Gigli and Savaré [4] among other authors. All this relied on Brenier’s characterization of optimal transport maps as gradients [22] and McCann’s displacement interpolations [23, 24].

After this numerous authors modified the JKO scheme to study different PDE by changing the functional to be minimized, but still using the Wasserstein distance (Santambrogio’s works [6] and [25, §8.4.2] contain excellent expositions). In work more relevant to ours, some authors have considered JKO schemes with different transport costs. These include Agueh [26] who considered transport costs

6 $$\begin{aligned} \mathcal {T}_c(\mu ,\nu ) = \inf _{\mu ,\nu \in \Pi (\mu ,\nu )} \int _{\textbf{R}^n \times \textbf{R}^n} c(x-y)\, \textrm{d}\pi (x,y), \end{aligned}$$

where $c(\cdot )$ is strictly convex and Figalli, Gangbo, and Yolcu [27] who considered an iteration scheme for cost functions of the form $c(x,y) = \inf L(\gamma ,\dot{\gamma })$, where the infimum is over differentiable curves $\gamma$ with endpoints $\gamma (0) = x$ and $\gamma (1) = y$ and *L* is a Lagrangian. Natile, Peletier, and Savaré [28] considered contractivity properties of (6) along solutions of the Fokker–Planck equation. Figalli and Gigli [29] modified the transport cost so as to consider the heat equation with Dirichlet boundary conditions. Finally Zhang [30], Erbar [31] and Savare [32] considered the JKO scheme with the Wasserstein distance on Riemannian manifolds with Ricci curvature bounded below. Recently, Deb et al. [33] introduced a notion of mirror Wasserstein gradient flow and characterized it as a scaling limit of the Sinkhorn algorithm.

The above works have informed ours, and have advantages and disadvantages over ours. Indeed, Agueh’s work as well as Figalli, Gangbo’s and Yolcu’s consider a broader class of PDE. However, their work takes place on Euclidean space whereas we work on Riemannian manifolds. In addition, the transport costs in the above works do not include our motivating example (3) (those in Agueh’s work are translation invariant and the Bregman divergence is not, in general, induced by a Lagrangian of the type considered in [27]).

Our paper is structured as follows. In Section 2 we give preliminaries from optimal transport and real analysis. In Section 3 we compute differentiation formulas for the entropy, drift and the transport cost $\mathcal {T}_c$. Then in Sections 4 and 5 we prove Theorem 1. Our proofs follow Figalli and Glaudo’s book [34] as well as Jordan, Kinderlehrer and Otto’s paper [1]. We use details in the Riemannian setting from Zhang [30] and Erbar [31]. The Bregman case is discussed in Section 6. In Section 7 we conclude and discuss some future directions. Auxiliary results about weak solutions of the Fokker–Planck equation are given in Appendix A.

**Table 1:** Notation used in the paper

Symbol	Meaning
$(N, g = \langle \cdot , \cdot \rangle )$	Ambient Riemannian manifold
*d*(*x*, *y*)	Riemannian distance on *N*
*M*	Open submanifold of *N* (either precompact or *N*)
$\nabla , \textrm{div}$	Riemannian gradient and divergence
$D_i, D_{ij}$	Euclidean partial derivatives (in coordinates)
$u_{x^i}, \dot{u}$	Partial derivative in coordinates and time derivative
$\mathcal {P}(M)$	Space of (Borel) probability measures on *M*
*c*(*x*, *y*)	Cost function which induces *g*
$\mathcal {T}_c, \mathcal {W}_2$	Optimal transport cost w.r.t. *c* and Wasserstein distance w.r.t. *d*

## Preliminaries

In this section we state required definitions, assumptions, and preliminary results. For general background in differential geometry and optimal transport we refer the reader to [4, 8, 35]. Throughout we adopt the summation convention.

### Preliminaries on optimal transport and the JKO scheme

Let (*N*, *g*) be an ambient Riemannian manifold with metric *g*. We denote the Riemannian distance on *N* by *d*(*x*, *y*) and the volume measure by $\textrm{dVol}_{g}$. Let $M \subset N$ be an open submanifold of *N*. When we are interested in the Neumann problem we take $\overline{M}$ to be compact with (nonempty) smooth boundary; when we are interested in the global problem we take $M = N$. As stated in the introduction, whilst one may regard the cost as inducing the Riemannian metric, it is equivalent to assume that a Riemannian metric is given and the cost agrees with it (in the sense of (2)). By a cost function on *N* we mean $c\in C^\infty (N \times N)$ satisfying $c(x,y) \ge 0$ with equality if and only if $x = y$. In statistics (see, e.g., [15]) such a function is sometimes called a divergence, or generalized distance, on *N*. We assume that *c* is compatible with *g* in the sense that (2) holds for any coordinate system (applied to both *x* and *y*). Since *c* vanishes on the diagonal of $N \times N$, we also have

$$ g_{ij}(x) = c_{x^i, x^j}(x, y)|_{x = y} = c_{y^i, y^j}(x, y)|_{x = y} = -c_{x^j, y^i}(x, y)|_{x = y}. $$

It can be shown that (2) implies the local approximation $c(x, y) \approx \frac{1}{2}d^2(x, y)$. For additional details and results see [36, Section 11.3] and [37]. While $c(x, y) = \frac{1}{2}d^2(x, y)$ recovers the metric via (2) and is currently the default choice,^1^ our approach, which is based on (2), allows a wide variety of cost functions which may be more suitable in particular applications.

We denote the space of Borel probability measures on *M* by $\mathcal {P}(M)$. We say that $\rho \in \mathcal {P}(M)$ has finite second moment if for some, and thus any, $x_0 \in M$ we have $\int _M d^2(x, x_0) \, \textrm{d}\rho (x) < \infty$. We are interested in Wasserstein gradient flows, and their approximation schemes, for the entropy functional

$$\begin{aligned} E(\mu ) = {\left\{ \begin{array}{ll} \int _{M} \log \rho \, \textrm{d}\rho & \text { if } \textrm{d}\mu (x) = \rho (x) \textrm{dVol}_{g}(x),\\ +\infty & \text { otherwise}. \end{array}\right. } \end{aligned}$$

The constant factor $\beta > 0$ in (5) refers to the inverse temperature and is immaterial in the mathematical analysis, so in the rest of the paper we fix once and for all that $\beta = 1$. We will identify an absolutely continuous probability measure with respect to $\textrm{dVol}_{g}$ with its density. Gradient flows of *E* yield the heat equation, but it is little extra work to couple the entropy with a drift term thereby obtaining the Fokker–Planck equation. Thus we define the drift term

7 $$\begin{aligned} D(\mu ) = \int _M \psi \, \textrm{d}\mu ,\end{aligned}$$

for $\psi \in C^\infty (M)$ a fixed nonnegative and bounded function satisfying the estimate

$$\begin{aligned} \Vert \nabla \psi (x)\Vert \le C(\psi (x)+1). \end{aligned}$$

Let $\mu ,\nu \in \mathcal {P}(M)$. The optimal transport cost with cost *c* is

$$\begin{aligned} \mathcal {T}_{c}(\mu ,\nu ) = \inf _{\pi \in \Pi (\mu ,\nu )}\int _{M \times M}c(x,y) \, \textrm{d}\pi (x,y), \end{aligned}$$

where $\Pi (\mu ,\nu )$ is the set of probability measures on $M \times M$ with marginals $\mu$ and $\nu$. When $c = B_{\varphi }$ is a Bregman divergence $\mathcal {T}_{c}(\mu ,\nu ) = \mathcal {B}_{\varphi }(\mu ,\nu )$ becomes the Bregman–Wasserstein divergence (4).

Let us now assume we have a probability measure $\rho _0 \in \mathcal {P}(M)$ with finite second moment and entropy. In particular, $\rho _0$ is absolutely continuous. For each $\tau > 0$ we recursively define

$$\begin{aligned} \rho ^\tau _{k+1} := \text {argmin}_{\rho \in \mathcal {P}(M)} \, E(\rho )+D(\rho ) + \frac{1}{\tau }\mathcal {T}_{c}(\rho ,\rho ^\tau _k). \end{aligned}$$

It is useful to set

8 $$\begin{aligned} J_k(\rho ) := E(\rho )+D(\rho ) + \frac{1}{\tau }\mathcal {T}_{c}(\rho ,\rho ^\tau _k). \end{aligned}$$

Provided this sequence is well defined, which we prove, one may define a piecewise constant curve of (absolutely continuous) probability measures by

$$\begin{aligned} \rho ^\tau (t) = {\left\{ \begin{array}{ll} \rho _0 &  \text { for }t=0,\\ \rho ^\tau _k & \text { for }t \in \big ((k-1)\tau ,k\tau \big ]. \end{array}\right. } \end{aligned}$$

Our main results, stated already in Theorems 1 and 2, are that these interpolants converge to solutions of the Fokker–Planck equation.

### Preliminary analysis

We will repeatedly consider sequences of probability densities and need to prove some subsequence converges in a suitable sense. We achieve this via the Dunford–Pettis theorem in the below form. For completeness, we also recall some standard definitions and results.

Let $(X, \mu )$ be a $\sigma$-finite measure space. We say that a sequence $(f_n)_{n \ge 1} \subset L^1(X; \mu )$ converges weakly (in $L^1$) to $f \in L^1(X; \mu )$, written $f_n \rightharpoonup f$, provided $\lim _{n \rightarrow \infty }\int _{X} f_n k \, \textrm{d}\mu = \int _{X} f k \, \textrm{d}\mu$ for all $k \in L^{\infty }(X; \mu )$. The Dunford-Pettis theorem (see e.g., [39, Lemma 5.13]) states that when $\mu (X) < \infty$, a collection $\mathcal {A} \subset L^1(X; \mu )$ is relatively weakly compact if and only if it is uniformly integrable, i.e., $\lim _{R \rightarrow +\infty } \sup _{f \in \mathcal {A}} \int _{\{|f_n| \ge R \}}|f_n| \, \textrm{d}\mu = 0$. We also recall that $\mathcal {A}$ is uniformly integrable if and only if there exists a superlinear function, namely $h: [0,\infty ] \rightarrow [0,\infty ]$ satisfying $\lim _{x \rightarrow +\infty }h(x)/x = +\infty$, such that $\sup _{f \in \mathcal {A}} \int _X h(|f_n|) \, \textrm{d}\mu < \infty$. Next, let *X* be a metric space and $(\rho _n)_{n \ge 1} \subset \mathcal {P}(X)$. We say that $\rho _n$ converges narrowly to $\rho \in \mathcal {P}(X)$ if $\lim _{n \rightarrow \infty }\int _{X} k \, \textrm{d}\rho _n = \int _{X} k \, \textrm{d}\rho$ for all bounded continuous functions *k* on *X*.

#### Lemma 3

Let (*X*, *d*) be a proper metric space, i.e., all closed balls are compact, and let $\mu$ be a Radon measure on *X*. Let $(f_n)_{n \ge 1} \subset L^1(X;\mu )$ be such that $f_n \ge 0$ and $f_n \, \textrm{d}\mu$ are probability measures, i.e., $\int _{X}f_n\, \textrm{d}\mu = 1$. Also suppose there exists a superlinear $h:[0,\infty ) \rightarrow [0,\infty )$ and $x_0 \in X$ such that

$$\begin{aligned} \sup _{n} \int _X h(f_n) \, \textrm{d}\mu&< +\infty , \text { and } \sup _{n} \int _X d^2(x,x_0) f_n \, \textrm{d}\mu < +\infty . \end{aligned}$$

Then the sequence has a weakly convergent subsequence $f_{k_j} \rightharpoonup f$ such that $\int _{X}f \, \textrm{d}\mu = 1$ and, in addition, $f_{k_j} \, d \mu$ converges narrowly to $f \, d \mu$.

#### Proof

For completeness we provide a sketch of the proof. The superlinearity estimate and our assumptions on (*X*, *d*) and $\mu$ allow us to apply Dunford–Pettis on each ball $B_N:= B(x_0, N)$ where $x_0$ is arbitrary and $n \in \textbf{N}$. Coupled with a diagonalization argument we obtain a limiting function $f \ge 0$ to which, up to a subsequence, $f_n \rightharpoonup f$ in $L^1(B_N)$ for every *N*. The second moment estimate implies the subsequence $f_n \, \textrm{d}\mu$ is tight as elements of $\mathcal {P}(X)$. Thus by Prokhorov’s theorem (see [40, Theorem 23.2]) it converges narrowly to some probability measure. Testing on each $B_N$ shows that the narrow limit is $f \textrm{d}\mu$. Now it is standard to show that $f_n \rightharpoonup f$ in $L^1(X; \mu )$. $\square$

### Lower semicontinuity and convexity of $J_k$

As a combination of standard results we obtain the following lemma.

#### Lemma 4

Provided $\rho ^{\tau }_k \in \mathcal {P}(M)$ satisfies $E(\rho ^{\tau }_k) < \infty$ and $D(\rho ^{\tau }_k) < \infty$, the functional $J_k$ on $\mathcal {P}(M)$ as defined in (8) is lower semicontinuous (with respect to narrow convergence) and strictly convex.

#### Proof

We provide references: The transport cost is lower semicontinuous and convex by [8, Lemma 4.3 and Theorem 4.8]. The lower semicontinuity of the entropy and drift follows from [4, Lemma 5.1.7] and [8, Theorem 29.20]. Finally, the drift term is linear, and the entropy is strictly convex. $\square$

### On *c*-convex sets and *c*-segments

When working with general cost functions certain assumptions are standard [8, 41]. Namely, we assume:

**A1. ** For all $x \in N$ the map $y \mapsto -D_{x}c(x,y)$ from *N* to $T_{x}^*N$ is injective. Similarly, for all $y \in N$ the map $x \mapsto -D_{y}c(x,y)$ from *N* to $T_{x}^*N$ is injective.

**A2. ** For each $(x,y) \in N^2$ and choice of coordinate systems about *x* and about *y*

$$\begin{aligned} \det c_{x^i,y^j}(x,y) \ne 0. \end{aligned}$$

As noted in [42] the condition A2 implies, via a topological argument, the manifold *N* is not compact. Indeed, in coordinates about any $x_0$ the image of *N* under the map $y \mapsto c_{x^{i}}(x_0,y)\partial _i$ is, by A2, an open subset of $T_{x_0}N \sim \textbf{R}^{n}$ and were *N* compact this image would also be compact — a contradiction.

A novel feature of our argument for dealing with general costs is estimates along *c*-segments. These are a class of curves, akin to line segments, introduced by Ma, Trudinger, and Wang [41] to study the regularity of optimal transport. In the Riemannian setting [8, Definition 12.10] the *c*-segment with respect to *x* joining $y_0$ to $y_1$ is the curve $(y_t)_{0 \le t \le 1}$ defined by

9 $$\begin{aligned} \nabla _{x}c(x,y_t) = (1-t)\nabla _{x}c(x,y_0) + t \nabla _{x}c(x,y_1). \end{aligned}$$

When such a curve exists it is unique by A1 and is smooth by A2. We also assume that for any $x, y_0, y_1 \in N$ there exists a *c*-segment with respect to *x* joining $y_0$ to $y_1$. That is, *N* is *c*-convex with respect to itself.

#### Lemma 5

(Initial velocity of *c*-segment) If $(y_t)_{0 \le t \le 1}$ is a *c*-segment with respect to *x* from *x* to *y*, then $\dot{y}_0 = -\nabla _{x}c(x,y)$.

#### Proof

Since $z \mapsto c(x,z)$ is minimized at *x*, $\nabla _x c(x, x) = 0$ and (9) reduces to

10 $$\begin{aligned} \nabla _x c(x, y_t) = t \nabla _x c(x, y_1), \quad 0 \le t \le 1. \end{aligned}$$

In a coordinate neighbourhood *U* about *x* we have

$$ c_{x^i}(x,y_t) = t c_{x^i}(x,y). $$

With *t* so small that $y_t \in U$, differentiating with respect to *t* implies

11 $$\begin{aligned} c_{x^i, y^j}(x, y_t) \dot{y}_t^j = c_{x^i}(x, y). \end{aligned}$$

When $t = 0$ then $y_0 = x$, and the identity $g_{ij}(x) = -c_{x^i, y^j}(x, x)$ implies

$$\begin{aligned} \dot{y}_0^k = g^{ki}(x)c_{x^i,y^j}(x,x)\dot{y}^j_0 = -g^{ki}(x,x)c_{x^i}(x,y). \end{aligned}$$

That is, $$\dot{y}_0 = -\nabla _{x}c(x,y)$$. $$\square $$

## Differentiation formulas

We collect in the following lemma some required differentiation formulas. We work under the setting of Section 2.1.

### Lemma 6

Assume either

(i) $\overline{M}$ is compact in *N* with (non-empty) smooth boundary, *c* is a cost function on *N*, and $\xi$ is a $C^{0,1}$ vector field on $\overline{M}$ tangential to $\partial M$, i.e., $\langle \xi , \textbf{n} \rangle = 0$ on $\partial M$ for $\textbf{n}$ the outer unit normal; or
(ii) $M = N$ and (*M*, *g*) is a complete Riemannian manifold, *c* is a cost function on *M*, and $\xi$ is a $C^{0, 1}$ vector field with compact support.

Then for $\Phi _t: M \rightarrow M$ the flow of $\xi$ (which is, for small time, a diffeomorphism), $\rho _0 \in \mathcal {P}(M)$ with finite entropy and second moment, $\rho _t:= (\Phi _t)_{\#} \rho _0$, and $\mu \in \mathcal {P}(M)$ with $\mathcal {T}_c(\rho _0, \mu ) < \infty$, the following differentiation formulas hold:

12 $$\begin{aligned} \frac{\textrm{d}}{\textrm{d}t} \Big \vert _{t = 0} D(\rho _t)&= \int _M \langle \nabla \psi , \xi \rangle \, \textrm{d}\rho _0, \end{aligned}$$

13 $$\begin{aligned} \frac{\textrm{d}}{\textrm{d}t} \Big \vert _{t = 0} E(\rho _t)&= -\int _{M} \textrm{div}\xi \, \textrm{d}\rho _0, \end{aligned}$$

14 $$\begin{aligned} \limsup _{t \rightarrow 0^+} \frac{1}{t}\left( \mathcal {T}_{c}(\rho _t,\mu ) - \mathcal {T}_{c}(\rho _0,\mu )\right)&\le \int _{M\times M} \langle \xi (x), \nabla _{x}c(x,y)\rangle \, \textrm{d}\pi (x,y), \end{aligned}$$

where $\pi \in \Pi (\rho _0, \mu )$ is optimal for the transport $\mathcal {T}_{c}(\rho _0,\mu )$.

### Proof

Equation (12) follows from the continuity equation and whilst (13) appears in the literature [31], for completeness we include a proof. Consider

15 $$\begin{aligned} E(\rho _t) = \int _{M} ( \log \rho _t)\rho _t \textrm{dVol}_{g}= \int _{M} \log (\rho _t(\Phi _t(x))) \textrm{d}\rho _0. \end{aligned}$$

The change of variables formula ([8, pg. 12]) implies

16 $$\begin{aligned} \rho _0(x) = \rho _t(\Phi _t(x))\mathcal {J}_t(x), \end{aligned}$$

for $\mathcal {J}_t$ the Jacobian determinant of $\Phi _t$. Let $\{U_{\ell }\}$ be a finite covering of the support of $\xi$ with coordinate neighbourhoods. In each neighbourhood

$$\begin{aligned} \mathcal {J}_t(x) = \lim _{\varepsilon \rightarrow 0} \frac{\text {Vol}_g(\Phi _t(B_\varepsilon (x)))}{\text {Vol}_g(B_\varepsilon (x))} = \frac{\sqrt{\det g_{ij}(\Phi _t(x))} \, | \!\det D\Phi _t|}{\sqrt{\det g_{ij}(x)}}. \end{aligned}$$

This gives

$$\begin{aligned} \int _{U_{\ell }} \log (\rho _t(\Phi _t(x))) \textrm{d}\rho _0 = \int _{U_{\ell }} \log \left( \frac{\rho _0(x)\sqrt{\det g_{ij}(x)}}{\sqrt{\det g_{ij}(\Phi _t(x))} |\det D\Phi _t|}\right) \textrm{d}\rho _0. \end{aligned}$$

Since $\xi \in C^{0, 1}$ differentiation under the integral is justified by the dominated convergence theorem. Thus, using $\text {tr}$ to denote the trace, we have

$$\begin{aligned}&\frac{\textrm{d}}{\textrm{d}t} \int _{U_{\ell }} \log (\rho _t(\Phi _t(x))) \rho _0 \textrm{dVol}_{g}\\&= - \int _{U_{\ell }} \frac{1}{\sqrt{\det g_{ij}(\Phi _t)}} \dot{\Phi }_t^k D_k \sqrt{\det g_{ij}(\Phi _t)} + \text {tr}\big ((D\Phi _t)^{-1}D\dot{\Phi }_t\big ) \textrm{d}\rho _0, \end{aligned}$$

Thus at time 0, when $\Phi _0, D\Phi _0 = \textrm{Id}$ and $\dot{\Phi }^k_0 = \xi ^k$, the product rule implies

17 $$\begin{aligned} -\int _{U_{\ell }} \frac{1}{\sqrt{\det g_{ij}}} D_k \Big ( \xi ^k \sqrt{\det g_{ij}}\Big ) \textrm{d}\rho _0 = -\int _{U_\ell } \textrm{div}\xi \, \textrm{d}\rho _0. \end{aligned}$$

The last equality uses the coordinate expression of the Riemannian divergence as in [43, Proposition 2.46]. From this we obtain (13) using a partition of unity subordinate to our coordinate neighborhoods.

To prove (14) let $\pi \in \Pi (\rho _0,\mu )$ be an optimal plan for $\mathcal {T}_c(\rho _0,\mu )$. Then $(\Phi _t, \textrm{Id})_{\#}\pi \in \Pi (\rho _t,\mu )$ and so is a sub-optimal plan for $\mathcal {T}_c(\rho _t,\mu )$. Thus,

$$\begin{aligned}&\mathcal {T}_{c}(\rho _t,\mu ) - \mathcal {T}_{c}(\rho _0,\mu )\\&\quad \le \int _{M \times M} c(x,y) \, \textrm{d}(\Phi _t,\textrm{Id})_{\#}\pi (x,y) - \int _{M \times M} c(x,y) \, \textrm{d}\pi (x,y) \\&\quad = \int _{M \times M} c(\Phi _t(x),y) - c(x,y) \, \textrm{d}\pi (x,y). \end{aligned}$$

Applying Taylor’s theorem to the function $t \mapsto c(\Phi _t(x),y)$ yields

$$\begin{aligned} \mathcal {T}_{c}(\rho _t,\mu ) - \mathcal {T}_{c}(\rho _0,\mu ) \le \int _{M \times M} t\langle \nabla _{x}c(x,y), \xi (x) \rangle + \frac{t^2}{2}R(\overline{t},x,y) \, \textrm{d}\pi (x,y), \end{aligned}$$

for some $\overline{t} \in [-|t|,|t|]$. Our assumption that either the space or the support of $\xi$ is compact along with $\xi \in C^{0,1}$ imply for $t > 0$ sufficiently small, $R(t,x, y) \le C$ for $C > 0$ independent of *x* and *y*. Thus, since $\pi$ is a probability measure, we obtain

$$\begin{aligned} \limsup _{t \rightarrow 0^+} \frac{1}{t}\left( \mathcal {T}_{c}(\rho _t,\mu ) - \mathcal {T}_{c}(\rho _0,\mu )\right) \le \int _M \langle \nabla _{x}c(x,y), \xi (x) \rangle \, \textrm{d}\pi (x,y). \end{aligned}$$

This is (14) and completes the proof. $\square$

## Pre-compact submanifolds

In this section we prove case (i) of Theorem 1. There are three key steps: the existence of $\rho ^\tau _{k+1}$ for $k=0,1,2,\dots$, a derivation of the minimality equation satisfied by $\rho ^\tau _{k+1}$, and finally the derivation of the Fokker–Planck equation. In the following all hypotheses of Theorem 1(i) are assumed.

### Existence of minimizer

Recall that $\rho _0$ is a density on *M* with finite entropy.

#### Lemma 7

For each $\tau > 0$ and $k = 0,1,2 \ldots$, the minimization problem (5) has a unique minimizer $\rho _{k+1}^\tau \in \mathcal {P}(M)$ which satisfies

18 $$\begin{aligned} E(\rho ^\tau _{k+1})+D(\rho ^\tau _{k+1}) \le \frac{1}{\tau }\mathcal {T}_{c}(\rho ^{\tau }_{{k+1}},\rho ^\tau _{k}) + E(\rho ^\tau _{k+1})+D(\rho ^\tau _{k+1}) \le E(\rho ^\tau _{k})+D(\rho ^\tau _k), \end{aligned}$$

in particular each $\rho ^\tau _k$ has finite entropy and drift.

#### Proof

By induction we prove the existence of $\rho _{k+1}^{\tau }$ when $\rho ^\tau _k$ is well defined. Recall $\rho ^\tau _{k+1} = \text {argmin}_{\rho \in \mathcal {P}(M)}J_k(\rho )$, where

$$\begin{aligned} J_k(\rho )=\int _{M} \log \rho \, \textrm{d}\rho + \int _{M} \psi \, \textrm{d}\rho + \frac{1}{\tau }\mathcal {T}_{c}(\rho ,\rho ^\tau _{k}). \end{aligned}$$

Since *M* is precompact in *N*, $\text {Vol}_g(M)$ is finite. Since also $\rho \log \rho \ge -1$ and $\psi \ge 0$, $J_k$ is bounded below. Thus we may take a minimizing sequence $\{\rho ^{\tau }_{k,i}\}_{i \ge 1}$ satisfying

$$\begin{aligned} \lim _{i \rightarrow \infty }J_k(\rho ^{\tau }_{k,i}) = \inf _{\rho \in \mathcal {P}(M)}J_k(\rho ). \end{aligned}$$

We assume, without loss of generality, that $C = \sup _i J_k(\rho ^{\tau }_{k,i}) < \infty$.

We extract a weakly convergent subsequence via Lemma 3. Indeed, since *M* is bounded we have trivially $\sup _i\int _M d^2(x, x_0) \textrm{d}\rho _{k, i}^{\tau } < \infty$. In addition,

$$\begin{aligned} C \ge J_k(\rho ^\tau _{k,i})&= \int _{M} \psi \, \textrm{d}\rho ^\tau _{k,i} + \int _{M} \log \rho ^\tau _{k,i} \, \textrm{d}\rho ^\tau _{k,i} + \frac{1}{\tau }\mathcal {T}_{c}(\rho ^\tau _{k,i},\rho ^\tau _{k})\\&\ge \int _M (\rho ^\tau _{k,i} \log \rho ^\tau _{k,i})_{+} \textrm{dVol}_{g}- \int _M (\rho ^\tau _{k,i} \log \rho ^\tau _{k,i})_{-} \textrm{dVol}_{g}\\&\ge \int _M (\rho ^\tau _{k,i} \log \rho ^\tau _{k,i})_{+} -\text {Vol}_{g}(M). \end{aligned}$$

Since $z \mapsto (z \log z)_+$ is superlinear, Lemma 3 applies. Thus up to a subsequence $\rho ^\tau _{k,i}$ converges narrowly to some absolutely continuous (thanks to finiteness of the entropy) probability measure $\rho ^\tau _{k+1}$ which, via the weak lower semicontinuity and strict convexity of $J_k$ (Lemma 4), is the unique minimizer.

To conclude the proof, we note since $\rho ^\tau _{k+1}$ minimizes $J_k$, (18) is simply a restatement of $J_k(\rho ^\tau _{k+1}) \le J_k(\rho ^\tau _k)$. $\square$

### Minimality condition

Using Lemma 6 it is straightforward to find the Euler–Lagrange equation satisfied by $\rho ^\tau _{k+1}$.

#### Lemma 8

Consider $\rho ^{\tau }_{k+1}$ defined by (5). Then for any $C^{0,1}(\overline{M})$ vector field $\xi$ tangential to $\partial M$ and $\pi$ an optimal plan for $\mathcal {T}_{c}(\rho ^\tau _{k+1},\rho ^\tau _k)$, we have

19 $$\begin{aligned} \int _{M}\textrm{div}\xi - \langle \nabla \psi , \xi \rangle \, \textrm{d}\rho ^\tau _{k+1} = \frac{1}{\tau }\int _{M \times M }\langle \xi (x), \nabla _{x}c(x,y) \rangle \, \textrm{d}\pi (x, y). \end{aligned}$$

#### Proof

As in Lemma 6 we let $\Phi _t$ denote the flow of $\xi$, and set^2^$\rho _t = (\Phi _t)_{\#} \rho ^\tau _{k+1}$. Minimality implies for $t > 0$,

$$\begin{aligned} 0 \le \frac{1}{t}\big (J_k(\rho _t) - J_k(\rho _0) \big ). \end{aligned}$$

Taking a limit supremum as $t \rightarrow 0^+$ and employing Lemma 6 yields

$$\begin{aligned} 0 \le - \int _{M} \textrm{div}\xi \, \textrm{d}\rho _0 + \int _M \langle \nabla \psi , \xi \rangle \, \textrm{d}\rho _0 + \frac{1}{\tau }\int _{M\times M}\langle \nabla _{x}c(x,y), \xi (x) \rangle \, \textrm{d}\pi (x,y). \end{aligned}$$

Since the same argument applies with $-\xi$ in place of $\xi$, we obtain the opposite inequality and subsequently (19). $\square$

### Derivation of the Fokker–Planck equation

Now we may complete the proof of Case (i) of Theorem 1.

#### Proof of Theorem 1(i)

*Step 1. (Weak convergence on* $[0,T] \times M$*)* As shown in Lemma 7, we have that $E(\rho ^\tau _k)+D(\rho ^\tau _k)$ is decreasing in *k*. The entropy is bounded below by $-\text {Vol}_{g}(M)$. Thus for any $T > 0$,

$$\begin{aligned} \int _0^T \int _{M} (\rho ^\tau \log \rho ^\tau )_{+} \, \textrm{dVol}_{g}\textrm{d}t \le T \left( E(\rho _0)+\text {Vol}(M)+\sup _M |\psi |\right) < \infty . \end{aligned}$$

Lemma 3 and a diagonalization argument yield $\rho :[0,\infty )\times M \rightarrow \textbf{R}$ and a subsequence, still denoted by $\rho ^{\tau }$, such that $\rho ^\tau$ converges to $\rho$ (narrowly and weakly) on $[0,T]\times M$ for every $T>0$.

To strengthen this to weak convergence for every *t*, we use that $\rho$ is smooth (in the sense of Lemma 12). This follows from what we now prove: that $\rho$ is the unique distributional solution of the Fokker–Planck equation. Uniqueness of the limit implies that convergence holds without taking subsequences.

*Step 2. (Derivation of distributional form of the Fokker–Planck equation)* Let $\zeta \in C^\infty (\overline{M})$ satisfy the Neumann condition $\langle \nabla \zeta (x), \textbf{n}(x)\rangle = 0$ on $\partial M$.

We aim to derive the discretized version of the Fokker–Planck equation ((25) below). Fix $x,y \in M$ and let $(y_t)_{0 \le t \le 1}$ be the *c*-segment with respect to *x* joining *x* to *y*, so that $\nabla _x c(x, y_t) = t \nabla _x c(x, y)$ by (10). Lemma 5 implies $\dot{y}_0 = - \nabla _x c(x, y)$. Taylor’s theorem applied to $h(t) = \zeta (y_t)$ yields

20 $$\begin{aligned} \zeta (y) - \zeta (x) + \langle \nabla _x c(x, y), \nabla \zeta (x) \rangle = R(x, y) := \int _0^1(1-t)h''(t) \, \textrm{d}t. \end{aligned}$$

We claim *R* satisfies an estimate

21 $$\begin{aligned} R(x, y) \le K c(x,y), \end{aligned}$$

for a constant $K > 0$ independent of $x,y \in M$. In coordinates about $y_t$ we have

22 $$\begin{aligned} h''(t) = \zeta _{kl}(y_t)\dot{y}^k_t\dot{y}^l_t +\zeta _{a}(y_t) \ddot{y}^a_t. \end{aligned}$$

Returning to (11) and differentiating again (though now *x*, *y* may be in separate coordinate neighbourhoods) we obtain

23 $$\begin{aligned} c_{x^i,y^jy^k}(x,y_t)\dot{y}^j_t\dot{y}^k_t + c_{x^i,y^j}(x,y_t)\ddot{y}^j_t = 0. \end{aligned}$$

Recalling $\dot{y}^{j}_t = c^{i,j}c_{x^i}(x,y)$ where $c^{i,j}$ denotes the inverse of $c_{x^i,y^j}$, and that $\zeta$ has compact support, we obtain from(22), (23), and condition A2 that

$$\begin{aligned} h''(t)&= \zeta _{kl}(y_t)\dot{y}^k_t\dot{y}^l_t -\zeta _{a}(y_t)c^{a,i}(x,y_t)c_{x^i,y^jy^k}(x,y_t)\dot{y}^j_t\dot{y}^k_t\\&\le K \Vert \nabla _{x}c(x,y)\Vert ^2, \end{aligned}$$

for a constant *K* initially depending on the choices of coordinate neighbourhoods about *x* and $y_t$. But since we may cover *M* by finitely many coordinate neighbourhoods, by taking the maximum of the resulting constants *K*, and noting that the definition of *h*(*t*) is coordinate independent, we obtain a finite constant *K* such that

$$ h''(t) \le K \Vert \nabla _x c(x, y) \Vert ^2, $$

for all $x, y \in M$. Finally, because $\nabla _{x}c(x,x) = 0$ and $y \mapsto \nabla _{x}c(x,y)$ is Lipschitz on *M* (uniformly in *x*), $\Vert \nabla _{x}c(x,y) \Vert \le K\, d(x,y)$. Our assumption $\lambda d^2(x,y) \le c(x,y)$ gives (21) and thus

24 $$\begin{aligned} |\zeta (y) - \zeta (x) + \langle \nabla _{x}c(x,y), \nabla \zeta (x) \rangle | \le K \, c(x,y). \end{aligned}$$

Integrating against an optimal plan for the transport $\mathcal {T}_{c}(\rho ^\tau _{k+1},\rho ^\tau _k)$ implies

$$\begin{aligned} \left| \int _{M \times M} \zeta (y) - \zeta (x) + \langle \nabla _{x}c(x,y) , \nabla \zeta (x) \rangle \, \textrm{d}\pi (x,y)\right| \le K \mathcal {T}_c(\rho ^\tau _{k+1},\rho ^\tau _k). \end{aligned}$$

Finally by the minimality condition, Lemma 8, applied with the tangential vector field $\xi = \nabla \zeta$ and the marginal condition we obtain

25 $$\begin{aligned} \left| \int _M \zeta \, \textrm{d}\rho ^\tau _{k} - \int _M \zeta \, \textrm{d}\rho ^\tau _{k+1} + \tau \int _M \Delta \zeta - \langle \nabla \psi , \nabla \zeta \rangle \, \textrm{d}\rho ^\tau _{k+1} \right| \le K \mathcal {T}_c(\rho ^\tau _{k+1},\rho ^\tau _k) . \end{aligned}$$

It is standard that the rest of conclusions of Theorem 1(i) follow from (25) (see, for example, [30]). For completeness we include the details.

Take nonnegative $\eta \in C^\infty _c([0,\infty ))$ so $\text {spt }\eta \subset [0,T)$ for some *T*. We simultaneously multiply (25) by $\eta (k\tau )$ and sum from $k=0$ to $k_0$ where $k_0\tau \ge T$ and obtain

26 $$\begin{aligned} \Big | \sum _{k=0}^{k_0} \, \eta (k\tau )\left( \int _{M} \zeta \, \textrm{d}\rho ^\tau _{k} -\int _{M} \zeta \, \textrm{d}\rho ^\tau _{k+1}\right) +&\int _{M}( \Delta \zeta - \langle \nabla \psi ,\nabla \zeta \rangle ) \tau \eta (k\tau ) \, \textrm{d}\rho ^\tau _{k+1} \Big | \nonumber \\&\le \tau C, \end{aligned}$$

where $C = C(\zeta ,c,\rho _0, \eta )$ is independent of $\tau$. Here we have used that $\sum _k \mathcal {T}_c(\rho ^\tau _{k+1},\rho ^\tau _k)$ is bounded by a telescoping sum; we defer the details to (31). We proceed to compute the terms in (26). First,

$$\begin{aligned} \sum _{k=0}^{k_0} \int _{M} \tau \eta (k\tau ) \Delta \zeta \, \textrm{d}\rho ^\tau _{k+1} = \sum _{k=0}^{k_0}\int _{M}\Delta \zeta \int _{k\tau }^{(k+1)\tau } \eta (k\tau ) \, \textrm{d}t \, \textrm{d}\rho ^\tau _{k+1}. \end{aligned}$$

If $t \in [k\tau ,(k+1)\tau ]$ we have $| \eta (k\tau ) - \eta (t)| \le C( \eta )\tau$ whereby

$$\begin{aligned} \sum _{k=0}^{k_0} \int _{M} \tau \eta (k\tau ) \Delta \zeta \, \textrm{d}\rho ^\tau _{k+1} = \sum _{k=0}^{k_0}\int _{M}\Delta \zeta \int _{k\tau }^{(k+1)\tau } \eta (t) \, \textrm{d}t \, \textrm{d}\rho ^\tau _{k+1} + O(\tau ). \end{aligned}$$

Where $O(\tau )$ denotes a quantity satisfying $|O(\tau )| \le C( \eta ,\zeta )\tau$. In addition, since $\rho ^\tau (t) = \rho ^\tau _{k+1}$ on $(k\tau ,(k+1)\tau ]$ we obtain

27 $$\begin{aligned} \sum _{k=0}^{k_0} \int _{M} \tau \eta (k\tau )\Delta \zeta \, \textrm{d}\rho ^\tau _{k+1} = \int _0^{\infty }\!\!\!\int _{M} \eta (t)\Delta \zeta \rho ^\tau \, \textrm{dVol}_{g}\, \textrm{d}t + O(\tau ). \end{aligned}$$

Similarly,

$$\begin{aligned} \sum _{k=0}^{k_0} \int _{M}\tau \eta (k\tau ) \langle \nabla \psi , \nabla \zeta \rangle \, \textrm{d}\rho ^\tau _{k+1} = \int _0^{\infty }\!\!\!\int _{M} \eta (t) \langle \nabla \psi , \nabla \zeta \rangle \rho ^\tau \, \textrm{dVol}_{g}\, \textrm{d}t + O(\tau ). \end{aligned}$$

Consider the remaining terms in (26). We compute

$$\begin{aligned}&\sum _{k=0}^{k_0} \eta (k\tau )\left( \int _{M} \zeta \, \textrm{d}\rho ^\tau _{k} -\int _{M} \zeta \, \textrm{d}\rho ^\tau _{k+1}\right) \\&\quad = \int _{M} \zeta \eta (0) \rho _0 \, \textrm{dVol}_{g}+\sum _{k=1}^{k_0}\int _{M} \zeta ( \eta (k\tau ) - \eta ((k-1)\tau )) \, \textrm{d}\rho ^\tau _{k}\\&\quad = \int _{M} \zeta \eta (0) \rho _0 \, \textrm{dVol}_{g}+ \sum _{k=1}^{k_0} \int _{M}\zeta \int _{(k-1)\tau }^{k\tau } \eta '(t) \, \textrm{d}t \, \textrm{d}\rho ^\tau _{k}. \end{aligned}$$

Then using again $\rho ^\tau = \rho ^\tau _k$ on $((k-1)\tau ,k\tau ]$ and $k_0\tau \ge T$ we have

28 $$\begin{aligned}&\sum _{k=0}^{k_0} \eta (k\tau )\left( \int _{M} \zeta \, \textrm{d}\rho ^\tau _{k} -\int _{M} \zeta \, \textrm{d}\rho ^\tau _{k+1}\right) \nonumber \\&\quad = \int _{M} \zeta \eta (0) \rho _0 \, \textrm{dVol}_{g}+ \int _{0}^{\infty }\int _{M} \eta '(t) \zeta \rho ^\tau \, \textrm{d}t \, \textrm{dVol}_{g}. \end{aligned}$$

Substituting (27) and (28) into (26) we obtain

$$ \left| \int _{M} \zeta \eta (0) \rho _0 \, \textrm{dVol}_{g}+ \int _{0}^\infty \int _{M} (\zeta \partial _t \eta + \eta (\Delta \zeta - \langle \nabla \psi ,\nabla \zeta \rangle ))\rho ^\tau \, \textrm{dVol}_{g}\textrm{d}t \right| \le C\tau . $$

On sending $\tau \rightarrow 0$ and using the weak $L^1_{\text {loc}}$ convergence we obtain the distributional form of the Fokker–Planck equation. Lemma 12 implies $\rho$ is the unique classical solution to the Fokker–Planck equation with Neumann boundary condition and initial condition $\rho _0$.

*Step 3. Weak* $L^1$ *convergence for every* *t*. Following [1] we strengthen the convergence to weak $L^1$ convergence for each *t*, that is for all $\zeta \in L^{\infty }(M)$

29 $$\begin{aligned} \lim _{\tau \rightarrow 0 }\int _{M} \zeta \, \textrm{d}\rho ^\tau (t) = \int _{M} \zeta \, \textrm{d}\rho (t). \end{aligned}$$

Indeed, it suffices to prove this for $\zeta \in C_c^{\infty }(M)$. Fix $\delta > 0$ and compute

30 $$\begin{aligned}&\Big \vert \int _M \zeta (x) \, \textrm{d}\rho ^\tau (t) -\int _M \zeta (x) \, \textrm{d}\rho (t )\Big \vert \nonumber \\&\le \Big \vert \int _M \zeta (x) \, \textrm{d}\rho ^\tau (t) - \frac{1}{2\delta }\int _{t-\delta }^{t+\delta }\int _M \zeta (x) \, \textrm{d}\rho ^\tau (r) \, \textrm{d}r \Big \vert \nonumber \\&\qquad +\Big \vert \frac{1}{2\delta }\int _{t-\delta }^{t+\delta }\int _M \zeta (x) \, \textrm{d}\rho ^\tau (r) \, \textrm{d}r - \frac{1}{2\delta }\int _{t-\delta }^{t+\delta }\int _M \zeta (x) \, \textrm{d}\rho (r) \, \textrm{d}r \Big \vert \nonumber \\&\qquad \qquad +\Big \vert \frac{1}{2\delta }\int _{t-\delta }^{t+\delta }\int _M \zeta (x) \, \textrm{d}\rho (r) \, \textrm{d}r - \int _M \zeta (x) \, \textrm{d}\rho (t) \Big \vert . \end{aligned}$$

We will send $\tau \rightarrow 0$ then $\delta \rightarrow 0$ to show that the limit is zero. Note the last term has limit 0 as $\delta \rightarrow 0$ because the solution of the Fokker–Planck equation is smooth. Namely the time derivative of $\rho$ is bounded on *M*^3^. On the other hand the second term tends to 0 as $\tau \rightarrow 0$ by the weak convergence coupled with the dominated convergence theorem.

We bound the first term in (30) by $C\sqrt{\delta +\tau }$ as follows. Using minimality (Lemma 7), a telescoping sum, and estimates for the drift and entropy we obtain

31 $$\begin{aligned} \frac{1}{\tau }\sum _{j=0}^k\mathcal {T}_c (\rho ^\tau _{j+1},\rho ^\tau _{j})&\le (D(\rho ^\tau _0)+E(\rho ^\tau _0))-(D(\rho ^\tau _{k+1})+E(\rho ^\tau _{k+1})) . \end{aligned}$$

Thus, using $D(\rho ^\tau _{k+1})+E(\rho ^\tau _{k+1}) \ge -\text {Vol}(M)$, $\sum _{k=0}^\infty \mathcal {T}_c (\rho ^\tau _{k+1},\rho ^\tau _{k}) \le C\tau .$ Assume $T \gg t$ is fixed and $N,N' \in \textbf{N}$ satisfy $N\tau ,N'\tau \le T$. We claim

32 $$\begin{aligned} \mathcal {T}_c(\rho ^\tau _{N'},\rho ^\tau _N) \le C\tau |N'-N|. \end{aligned}$$

Indeed, we invoke the triangle inequality by passing through the Wasserstein distance and obtain (assuming $N'>N$)

$$\begin{aligned} \mathcal {T}_c(\rho ^\tau _{N'},\rho ^\tau _N)&\le C \mathcal {W}_2^2(\rho ^\tau _{N'},\rho ^\tau _N)\\&\le C\left( \sum _{k=N}^{N'-1}\mathcal {W}_2(\rho ^\tau _{k+1},\rho ^\tau _{k})\right) ^{2}\\&\le C (N'-N)\sum _{k=N}^{N'-1}\mathcal {W}_2^2(\rho ^\tau _{k+1},\rho ^\tau _{k})\\&\le C(N'-N)\sum _{k=N}^{N'-1}\mathcal {T}_{c}(\rho ^\tau _{k+1},\rho ^\tau _{k}). \end{aligned}$$

The telescoping sum (31) yields (32).

In addition for arbitrary probability densities $\rho ',\rho$ we claim

33 $$\begin{aligned} \left| \int _M \zeta \, \textrm{d}\rho ' - \int _M \zeta \, \textrm{d}\rho \right| ^2 \le C(|\sup D\zeta |) \mathcal {T}_{c}(\rho ,\rho '). \end{aligned}$$

Indeed if $\pi$ is an optimal transport plan for $\mathcal {T}_{c}(\rho ,\rho ')$, we have

$$\begin{aligned} \begin{aligned}&\left| \int _M \zeta \, \textrm{d}\rho ' - \int _M \zeta \, \textrm{d}\rho \right| ^2 = \left| \int _M \zeta (y) - \zeta (x) \, \textrm{d}\pi (x,y) \right| ^2 \\&\quad \le \sup |D\zeta |^2 \frac{1}{\lambda } \int _M c(x,y) \textrm{d}\pi (x,y), \end{aligned} \end{aligned}$$

this last term is bounded by $\mathcal {T}_{c}( \rho ,\rho ')$. Thus, for any $t,t' \le T$ we may choose $N,N'$ such that $t \in ((N-1)\tau ,N\tau ]$ and similarly for $N'$. Then by (32)

$$\begin{aligned} \mathcal {T}_{c}(\rho ^\tau (t),\rho ^\tau (t')) =\mathcal {T}_{c}(\rho ^\tau _{N'},\rho ^\tau _N) \le C\tau |N-N'|. \end{aligned}$$

Which combined with (33) implies

34 $$\begin{aligned} \nonumber \left| \int _M \zeta \, \textrm{d}\rho ^\tau (t') - \int _M \zeta \, \textrm{d}\rho ^\tau (t)\right| ^2&\le C(\sup |D\zeta |)\tau |N-N'|\\&\le C(\sup |D\zeta |)(|t-t'|+\tau ). \end{aligned}$$

The latter inequality accounts for the fact that $t,t'$ may lie inside the intervals $(N\tau ,(N+1)\tau ]$, $(N'\tau ,(N'+1)\tau ]$. Thus using (34) the term (30) is bounded by $C\sqrt{\tau +\delta }$. This establishes (29) and completes the proof. $\square$

## Complete manifold

In this section we remove the compactness requirements (and the Neumann boundary condition) of the previous section and prove Case (ii) of Theorem 1. For this case $M = N$ and (*M*, *g*) is a complete Riemannian manifold with $\lambda d^2 \le c \le \Lambda d^2$ and Ricci curvature bounded below. By the Bishop–Gromov inequality (which we take from Erbar’s paper [31, Proof of Lemma 4.1]), there is *K* depending on the lower bound for the Ricci curvature satisfying

35 $$\begin{aligned} \int _{M}e^{ -d(x,x_0)/2} \textrm{dVol}_{g}(x) \le K < +\infty , \end{aligned}$$

where here and throughout $x_0$ denotes a fixed, but arbitrary, element of *M*. Inequality (35) is the key way in which the condition of Ricci curvature bounded below enters the proof. In this section we largely follow the original paper of Jordan, Kinderlehrer, and Otto [1].

### Existence of a minimizer

This step differs most from the compact case. The entropy is not bounded below and thus, apriori, $J_k$ (which we wish to minimize) may not be bounded below. To overcome this, we establish that $\mathcal {T}_c$ dominates the negative part of the entropy. We let $m(\rho ) = \int _{M} d^2(x,x_0)\ \, \textrm{d}\rho$ be the second moment of $\rho$ (about $x_0$) and establish the following estimates.

#### Lemma 9

Under the assumptions of Theorem 1 Case (ii) and with $\rho _0,\rho _1$ any nonnegative probability densities we have the following estimates.

(i) For all $\varepsilon > 0$ and $M' \subset M$ there holds
  36 $$\begin{aligned} \int _{M'} (\rho \log \rho )_{-} \le \int _{M'} e^{-d(x,x_0)/2} \, \textrm{dVol}_{g}+ \varepsilon m(\rho ) + \frac{1}{4\varepsilon } \int _{M'} \rho \, \textrm{dVol}_{g}. \end{aligned}$$
(ii) The optimal transport cost satisfies
  37 $$\begin{aligned} \mathcal {T}_c(\rho _0,\rho _1) \ge \frac{\lambda }{2} m(\rho _0) - \lambda m(\rho _1), \end{aligned}$$
  where $\lambda > 0$ is from the statement of Theorem 1.
(iii) There holds
  38 $$\begin{aligned} J_k(\rho ) \ge \frac{\lambda }{4\tau }m(\rho ) - \frac{\lambda }{\tau } m(\rho ^\tau _k)-C(K,\lambda ,\tau ). \end{aligned}$$

#### Proof

To prove (36) note if $z \in (0,1]$ then $(z \log z)_{-} = z |\log z| \le z^{1/2}$. Let

$$\begin{aligned} M_0&= \{x \in M'; \rho (x) \le e^{-d(x,x_0)}\text { and }\rho (x) \le 1\},\\ \text { and } M_1&= \{x \in M' ; e^{-d(x,x_0)} \le \rho (x)\text { and }\rho (x) \le 1\}. \end{aligned}$$

Then

$$\begin{aligned} \int _{M'} (\rho \log \rho )_{-}\textrm{dVol}_{g}&= \int _{M_0}(\rho \log \rho )_{-}\textrm{dVol}_{g}+ \int _{M_1} (\rho \log \rho )_{-}\textrm{dVol}_{g}\\&\le \int _{M_0} \rho ^{1/2} \textrm{dVol}_{g}+ \int _{M_1} \rho d(x,x_0) \textrm{dVol}_{g}\\&\le \int _{M'} e^{-d(x,x_0)/2} \textrm{dVol}_{g}+ \int _{M'} \varepsilon d^2(x,x_0) \rho +\frac{\rho }{4\varepsilon } \textrm{dVol}_{g}. \end{aligned}$$

We’ve used $ab \le \varepsilon a^2 + b^2/4\varepsilon$ with $a = d(x,x_0)$, $b=1$ and this proves (36).

To prove (37) take $\rho _0,\rho _1 \in \mathcal {P}(M)$ and let $\pi$ be an optimal plan for $\mathcal {T}_c(\rho _0,\rho _1)$. Then by the condition $\lambda d^2(x,y) \le c(x,y)$

39 $$\begin{aligned} \mathcal {T}_c(\rho _0,\rho _1) = \int _{M}c(x,y) \textrm{d}\pi (x,y) \ge \lambda \int _{M}d^2(x,y) \textrm{d}\pi (x,y). \end{aligned}$$

The triangle inequality implies $d^2(x,y) \ge d^2(x,x_0)/2 - d^2(x_0,y),$ whereby the marginal condition for $\pi$ implies (39) becomes

$$\begin{aligned} \mathcal {T}_c(\rho _0,\rho _1) \ge \frac{\lambda }{2}m(\rho _0) - \lambda m(\rho _1). \end{aligned}$$

For (38) we use $\psi \ge 0$, (36) with $\varepsilon = \lambda /(4\tau )$, and (37) to obtain

$$\begin{aligned} J_k(\rho )&= \frac{1}{\tau }\mathcal {T}_c(\rho ,\rho ^\tau _{k}) + \int _{M} \psi \, \textrm{d}\rho + \int _{M} \log \rho \, \textrm{d}\rho \\&\ge \frac{1}{\tau }\mathcal {T}_c(\rho ,\rho ^\tau _{k}) - \int _{M} (\rho \log \rho )_{-} \textrm{dVol}_{g}\\&\ge \frac{\lambda }{2\tau }m(\rho ) - \frac{\lambda }{\tau } m(\rho ^\tau _k) - \int _{M} e^{-d(x,x_0)/2} \, \textrm{dVol}_{g}\\&\quad \quad - \frac{\lambda }{4\tau } m(\rho ) - \frac{\tau }{\lambda } \int _{M} \rho \, \textrm{dVol}_{g}\\&\ge \frac{\lambda }{4\tau }m(\rho )- \frac{\lambda }{\tau } m(\rho ^\tau _k) - C(K,\lambda ,\tau ). \end{aligned}$$

This is (38) and completes the proof. $\square$

#### Lemma 10

Under the assumptions of Theorem 1 (ii) and for each $k=1,2,\dots$ the minimization problem (5) has a unique minimizer $\rho ^\tau _{k+1}$.

#### Proof

It is immediate from (38) that $J_k$ is bounded below (in this proof $\tau ,k$ are fixed). Because $J_k$ is bounded below we may take a minimizing sequence of probability densities $\rho ^\tau _{k,i}$ satisfying

40 $$\begin{aligned} \lim _{i\rightarrow \infty }J_k(\rho ^\tau _{k,i}) = \text {inf}_{\rho \in \mathcal {P}(M)}J_k(\rho ). \end{aligned}$$

Without loss of generality $\sup _{i}J_k(\rho ^\tau _{k,i}) < \infty$. To obtain a convergent subsequence by Lemma 3 it suffices to establish

41 $$\begin{aligned} \sup _{i}\int (\rho ^\tau _{k,i}\log \rho ^\tau _{k,i})_+ \textrm{dVol}_{g}< +\infty \end{aligned}$$

and a second moment bound. To this end, note from (38) we have

42 $$\begin{aligned} \sup _{i}m(\rho ^\tau _{k,i}) < +\infty . \end{aligned}$$

Combined with

$$\begin{aligned} J_k(\rho ) \ge \frac{1}{\tau }\mathcal {T}_c(\rho ,\rho ^\tau _k)+\int (\rho \log \rho )_{+}\textrm{dVol}_{g}- \int (\rho \log \rho )_{-} \textrm{dVol}_{g}, \end{aligned}$$

into which we substitute (37) and (36), we obtain (41). We conclude the existence of a subsequence $\rho ^\tau _{k,i_j}$ converging weakly to some $\rho ^\tau _{k+1}$.

Finally, it is again immediate by the lower semicontinuity and strict convexity of $J_k$ (Lemma 4) that $\rho ^\tau _{k+1}$ uniquely minimizes $J_k$. $\square$

The minimality equation for $\rho ^{\tau }_{k+1}$ is unchanged once we take compactly supported $\zeta$ and, once more, follows immediately from Lemma 6.

#### Lemma 11

Let $\rho ^\tau _{k+1}$ be the minimizer of the functional $J_k$. Then for every compactly supported Lipschitz vector field $\xi$ there holds

43 $$\begin{aligned} \int _{M} \textrm{div}\xi - \langle \nabla \psi , \xi \rangle \, \textrm{d}\rho ^\tau _{k+1} = \frac{1}{\tau }\int _{M \times M} \langle \xi (x), \nabla _{x}c(x,y) \rangle \textrm{d}\pi (x,y), \end{aligned}$$

where $\pi$ is an optimal plan for $\mathcal {T}_{c}(\rho ^\tau _{k+1},\rho ^\tau _{k})$.

### Derivation of the Fokker–Planck equation

#### Proof. (Theorem 1, Case (ii))

*Step 1. (Derivation of time discretized equation)* We explain the modifications needed to obtain (25) and the weak $L^1$ convergence on $[0,\infty ) \times M$. With these in hand, the derivation of the Fokker–Planck equation and improvement to weak $L^1$ convergence on *M* for each *t* follows verbatim Case (i). Thus, let $\zeta \in C^\infty _c(M)$ be a test function.

The compact support of $\zeta$ implies that by arguing as in (24) for all $x,y \in \text {spt }\zeta$ there holds

44 $$\begin{aligned} |\zeta (y) - \zeta (x) - \langle \nabla \zeta (x), \nabla _xc(x,y)\rangle | \le C(\zeta )d^2(x,y). \end{aligned}$$

This inequality clearly holds when neither *x* nor *y* is in $\text {spt }\zeta$. When *x* is in the support of $\zeta$ but *y* is not, we argue as follows. Let $\gamma _t$ be the (Riemannian) geodesic with $\gamma _0 = y$ and $\gamma _1 = x$ and set $f(t) = \zeta (\gamma _t) + \langle \nabla \zeta (\gamma _t), \nabla _xc(x,y)\rangle$. Let *T* be the supremum of *t* such that $\gamma _t$ is not in $\text {spt}\zeta$. Then, noting $\nabla \zeta (\gamma _T) = 0$ and thus $f(T) = 0$ we have

$$\begin{aligned} |\zeta (x) +&\langle \nabla \zeta (x), \nabla _xc(x,y)\rangle | \\&= |\zeta (x) + \langle \nabla \zeta (x), \nabla _xc(x,y)\rangle - \zeta (\gamma _T) - \langle \nabla \zeta (\gamma _T), \nabla _xc(x,y)\rangle |\\&= |f(1)-f(T)| \\&= |f'(T)(1-T) +(1-T)^2f''(t)/2| \le C d^2(x,y). \end{aligned}$$

for some $t \in [T,1]$. The final equality uses the calulation $f'(T) = \langle \text {Hess} \zeta (\gamma _t) \, \dot{\gamma _t}, \nabla _xc(x,y)\rangle$, $|\nabla _xc(x,y)| \le Cd(x,y)$ since $\nabla _xc$ is Lipschitz, and $|\frac{\textrm{d}^2}{\textrm{d}t^2}f(\gamma _t)| \le C|\dot{\gamma _t}|^2$ with $\gamma _t$ a constant speed geodesic whence $|\dot{\gamma }_t| = d(x,y)$. A similar, but simpler, estimate yields (44) for $y \in \text {spt }\zeta$ and $x \not \in \text {spt }\zeta$. Thus (44) holds for all $x,y \in M$.

We let $\pi$ denote an optimal plan for $\mathcal {T}_c(\rho ^\tau _{k+1},\rho ^\tau _k)$ and integrate (44) with respect to $\pi$. Using $\lambda d^2(x,y) \le c(x,y)$, we have

$$ \left| \int _{M\times M} \zeta (y) - \zeta (x) -\langle \nabla \zeta (x), \nabla _xc(x,y)\rangle \, \textrm{d}\pi (x,y) \right| \le C \, \mathcal {T}_c(\rho ^\tau _{k+1},\rho ^\tau _k).$$

Combined with the minimality equation (Lemma 11 with $\xi = \nabla \zeta$) and the marginal condition we obtain

45 $$\begin{aligned} \begin{aligned}&\left| \int _{M} \zeta \, \textrm{d}\rho ^\tau _{k} - \int _{M} \zeta \, \textrm{d}\rho ^\tau _{k+1} +\tau \int _{M} \Delta \zeta - \langle \nabla \psi , \nabla \zeta \rangle \, \textrm{d}\rho ^\tau _{k+1} \right| \\&\quad \le C \, \mathcal {T}_c(\rho ^\tau _{k+1},\rho ^\tau _k). \end{aligned} \end{aligned}$$

The remainder of the proof concerns the convergence as $\tau \rightarrow 0.$

*Step 2: Convergence estimates.* Now we establish the necessary estimates to apply Lemma 3 and conclude the existence of a weakly/narrowly convergent subsequence of $\rho ^\tau$ on $[0,\infty )\times M$. Fix $T>0$, we show there is *C* depending on *T* such that whenever $k_0\tau \le T$ there holds

46 $$\begin{aligned} m(\rho ^\tau _k)&\le C, \end{aligned}$$

47 $$\begin{aligned} \int (\rho ^\tau _k\log \rho ^\tau _k)_{+} \textrm{dVol}_{g}&\le C. \end{aligned}$$

We begin by showing $J_k(\rho _{k+1})$ is nonincreasing in *k*, and computing a telescoping sum we’ll use repeatedly. First, the minimality of $\rho ^\tau _{k+1}$ implies

48 $$\begin{aligned} \frac{1}{\tau }&\mathcal {T}_c(\rho ^\tau _{k+1},\rho ^\tau _{k}) + E(\rho ^\tau _{k+1}) + D(\rho ^\tau _{k+1}) = J_k(\rho ^\tau _{k+1}) \le J_k(\rho ^\tau _{k}) \end{aligned}$$

49 $$\begin{aligned}&= E(\rho ^\tau _{k}) + D(\rho ^\tau _{k}) \le J_{k-1}(\rho ^\tau _{k}). \end{aligned}$$

From this we extract

$$\begin{aligned} \frac{1}{\tau }\mathcal {T}_c(\rho ^\tau _{k+1},\rho ^\tau _{k}) \le E(\rho ^\tau _{k}) + D(\rho ^\tau _{k}) - E(\rho ^\tau _{k+1}) - D(\rho ^\tau _{k+1}), \end{aligned}$$

which we sum to obtain

50 $$\begin{aligned} \frac{1}{\tau }\sum _{j=0}^{k-1}\mathcal {T}_c(\rho ^\tau _{j+1},\rho ^\tau _{j}) \le E(\rho ^\tau _{0}) + D(\rho ^\tau _{0}) - E(\rho ^\tau _{k}) - D(\rho ^\tau _{k}). \end{aligned}$$

To obtain (46) we pass through the Wasserstein distance so that, at the expense of a constant, we may use the triangle inequality. By (37), we have

$$\begin{aligned} m(\rho ^\tau _k)&\le \frac{2}{\lambda }\mathcal {T}_c(\rho ^\tau _k,\rho ^\tau _0) + 2m(\rho ^\tau _0)\\&\le \frac{2\Lambda }{\lambda }\mathcal {W}_2^2(\rho ^\tau _k,\rho ^\tau _0) + 2m(\rho ^\tau _0)\\&\le k\frac{2\Lambda }{\lambda }\sum _{j=0}^{k-1}\mathcal {W}_2^2(\rho ^\tau _{j+1},\rho ^\tau _j)+2m(\rho ^\tau _0)\\&\le k\frac{2\Lambda }{\lambda ^2}\sum _{j=0}^{k-1}\mathcal {T}_c(\rho ^\tau _{j+1},\rho ^\tau _j)+2m(\rho ^\tau _0). \end{aligned}$$

Employing (50) we have

$$\begin{aligned} m(\rho ^\tau _k) \le (\tau k) \frac{2\Lambda }{\lambda ^2}\left( E(\rho ^\tau _{0}) + D(\rho ^\tau _{0}) - E(\rho ^\tau _{k})\right) +2m(\rho ^\tau _0). \end{aligned}$$

We’ve used $-D(\rho ^\tau _{k+1}) \le 0$. Next, $\tau k_{0} \le T$ and (36) with $\varepsilon = \lambda ^2/(4\Lambda T)$ gives

$$\begin{aligned} m(\rho ^\tau _k) \le C(\rho _0,K,\lambda ,\Lambda ,T) + \frac{1}{2} m(\rho ^\tau _k), \end{aligned}$$

which is (46). Finally, for (47) note

$$\begin{aligned} \int (\rho ^\tau _k\log \rho ^\tau _k)_{+} \textrm{dVol}_{g}\le J_{k-1}(\rho ^\tau _k) + \int (\rho ^\tau _k\log \rho ^\tau _k)_{-} \textrm{dVol}_{g}. \end{aligned}$$

Estimates for $J_{k-1}(\rho ^\tau _k)$ are straight forward since this quantity is nonincreasing. In addition (36) combined with (46) yields (47). We note also that the entropy and drift permit straightforward estimates (as required by Lemma 13) once (46) and (47) are obtained (see [1] for details).

We integrate (46) and (47) to obtain there is a curve $\rho (t)$ such that as $\tau \rightarrow 0$, at least up to a subsequence $\rho ^\tau (t) \rightarrow \rho (t)$ weakly on $[0,T ]\times M$ and by diagonalization we obtain a weak/narrow limit on $[0,\infty )\times M$.

Having obtained weak convergence and equation (45) the conclusions of Theorem 1 Case (ii) follow essentially verbatim the proof of Theorem Case (i) (from the point equation (25)). Note Lemma 13, which is where the condition $\Vert \nabla \psi \Vert \le C(1+\psi )$ is employed, yields the corresponding distributional form of the heat equation.

## The Bregman case

In this section we specialize to the Bregman case (Theorem 2). Let $\varphi$ be a smooth function defined on an open convex subset *N* of $\textbf{R}^n$, such that $D^2 \varphi (x)$ is positive definite for $x \in N$. In particular, $\varphi$ is strictly convex. As in (3), let the cost function *c*(*x*, *y*) be the Bregman divergence:

$$ c(x, y) = B_{\varphi }(x, y) = \varphi (x) - \varphi (y) - D\varphi (y) \cdot (x - y). $$

A direct computation (under the Euclidean coordinate system) gives

51 $$\begin{aligned} c_{x^i}(x, y) = D_i\varphi (x) - D_i\varphi (y), \quad c_{y^i}(x, y) = \varphi _{y^i y^k}(y) (x^k - y^k), \end{aligned}$$

and

52 $$\begin{aligned} c_{x^i, y^j}(x, y) = - D_{ij} \varphi (y). \end{aligned}$$

Setting $x = y$ in the last equation shows that *c* induces, in the sense of (2), the Hessian metric corresponding to the convex potential $\varphi$.^4^ On the other hand, writing

53 $$\begin{aligned} \begin{aligned} c(x, y)&= \frac{1}{2}|x - D\varphi (y)|^2 + \left( \varphi (x) - \frac{1}{2}|x|^2\right) \\&\quad - \left( \varphi (y) + \frac{1}{2}|D\varphi (y)|^2 - y \cdot D\varphi (y) \right) \end{aligned} \end{aligned}$$

shows that the Bregman cost is, up to additive terms of the form $h(x) + k(y)$, Euclidean in the mixed coordinate system $(x, D\varphi (y))$ on $N \times N$. See [14], and the references therein, for the properties and applications of the Bregman–Wasserstein divergence $\mathcal {B}_{\varphi }$ defined by (4).

Since $D^2 \varphi$ is positive definite, $D\varphi$ is a diffeomorphism.^5^ It follows from (51) and (52) that *c* satisfies conditions A1 and A2. From (51), we see that $y_t$ is a *c*-segment from $y_0$ to $y_1$ if and only if

$$ D \varphi (y_t) = (1 - t) D \varphi (y_0) + t D \varphi (y_1). $$

Thus *N* is *c*-convex with respect to itself if and only if $D \varphi (N)$ is convex. Note that $D \varphi (N)$ is convex if $(N, \varphi )$ is a convex function of Legendre type. Unfortunately, $(N, \varphi )$ being of Legendre type is not sufficient to yield the other conditions required in Theorem 2 which employs Theorem 1.

### Proof of Theorem 2

From the above discussion we see that under the assumptions of the theorem, the Bregman cost satisfies A1 and A2, and *N* is *c*-convex with respect to itself.

Consider Case (i). Since *M* is precompact, there exists constants $c_0, c_1 > 0$ such that $c_0 I \le D^2 \varphi \le c_1 I$. From this it is easy to see that

$$ c(x, y) = B_{\varphi }(x, y) \asymp |x - y|^2 \asymp d^2(x, y), \quad x, y \in M. $$

Thus Theorem 1(i) applies. Clearly Case (ii) is a special case of Theorem 1(ii).

### Example 1

Let $M = \textbf{R}^n$ and let $\varphi$ be a smooth convex function on $\textbf{R}^n$ such that $c_0I \le D^2 \varphi \le c_1I$ for some $c_0, c_1 > 0$. Let *g* be the Hessian metric $g = D^2 \varphi$. From standard comparison results, we see that (*M*, *g*) satisfies the assumptions of Theorem 2(ii). Apart from the Bregman divergence, another explicit cost function is the (modified) Mahalanobis distance defined by

$$ c(x, y) = \frac{1}{2}(x - y)^{\top } D^2 \varphi (y) (x - y). $$

Nevertheless, we note that Brenier’s theorem does not apply to this cost. Also see [45] for the special case where $D^2 \varphi$ is diagonal.

### Example 2

(Fisher-Rao geometry of multivariate Gaussian distributions) For $n \ge 1$, let $\mathcal {S}_+^n$ be the space of $n \times n$ positive definite matrices. Let $N = \textbf{R}^n \times \mathcal {S}_+^n$ which we identify with the space of multivariate Gaussian distributions on $\textbf{R}^n$ (i.e., $(m, \Sigma ) \leftrightarrow \mathcal {N}(m, \Sigma )$).^6^ Consider the metric *g* whose line element is given by

$$ \textrm{d}s^2 = (\textrm{d}m)^{\top } \Sigma ^{-1} (\textrm{d}m) + \frac{1}{2} \textrm{tr} \left( (\Sigma ^{-1} \textrm{d}\Sigma )^2\right) . $$

It is the Fisher–Rao information metric on the space of Gaussian distributions [46]. The authors of [46] derived explicit solutions to the Riemannian geodesic equation, but analytic formulas for the Riemannian distance (which requires integrating along the geodesic) are only available in special cases.

Consider the alternative parametrization

$$ \theta = (\theta _1, \theta _2) = \left( \Sigma ^{-1} \mu , \frac{-1}{2} \Sigma ^{-1} \right) , $$

which takes values in $\textbf{R}^n \times (-\mathcal {S}_+^n)$.^7^ Consider

$$ \varphi (\theta ) = -\frac{1}{4} \theta _1^{\top } \theta _2^{-1} \theta _1 - \frac{1}{4} \log \det (-2 \theta _2), $$

which corresponds to the log-partition function of $N(m, \Sigma )$. Then $\varphi$ is smooth and convex, and its Bregman divergence (which can be expressed in closed form) induces *g*. Thus Theorem 2(i) can be applied on precompact open submanifolds of *N*. Analogous statements can be made on any regular exponential family [47] on which a convex potential induces the Fisher-Rao metric.

## Conclusion and future directions

In this paper we propose and prove the convergence of a modified JKO scheme for the Fokker-Planck equation on a Riemannian manifold, where the (squared) Wasserstein distance is replaced by an optimal transport cost whose cost function is compatible with the underlying metric. This is not only of theoretical interest but also allows us to use cost functions, and hence transport costs, which are computationally more tractable than the Riemannian distance and Wasserstein distance. As a specific example, we consider the Bregman divergence which is a tractable cost on a Hessian Riemannian manifold induced by a convex potential. The following directions arise naturally:

(i) Computational algorithms for implementing the modified JKO scheme. In the Euclidean case, one way to implement the JKO step (JKO) is to use input convex neural networks [48] (which approximate the Brenier potential), possibly with entropic regularization (see [49–51] and the references therein). Since the optimal transport for a Bregman cost becomes Euclidean after a coordinate transformation, we expect that similar techniques can be applied to implement Wasserstein gradient flows of measures over a Hessian manifold. (We note that an implementation for a specific diagonal metric is given in [45].)
(ii) Investigation of other tractable costs. One of the main advantages of the Euclidean quadratic cost $|x - y|^2$ is Brenier’s theorem which characterizes the optimal transport map as a convex gradient. For general costs, when the optimal transport map exists it is given as a *c*-gradient of a suitable *c*-convex potential. Whilst *c*-convexity is generally abstract and not easy to work with, for some cost functions *c*-convexity can be expressed in terms of ordinary convexity. For example, consider the Dirichlet optimal transport problem [52] on the open unit simplex $\Delta _n = \{ p = (0, 1)^n: \sum _i p^i = 1\}$ with cost function
  54 $$\begin{aligned} c(p, q) = \log \left( \sum _{i = 1}^n \frac{1}{n} \frac{q^i}{p^i} \right) - \sum _{i = 1}^n \frac{1}{n} \log \frac{q^i}{p^i}. \end{aligned}$$
  It can be shown that the Kantorovich potential can be expressed in terms of a concave function on $\Delta _n$ and hence the techniques in (i) and [14] may apply.^8^ Also see [53] which generalizes (54) to a one-parameter family of logarithmic costs.
(iii) Relaxing technical conditions and extending to other PDEs. To establish convergence of the JKO scheme we impose several technical conditions. Some of these, including the two-sided comparison between *c*(*x*, *y*) and $d^2(x, y)$ as well as A2, are restrictive,^9^ so one may ask if they can be relaxed. Also, in this paper we consider only the Fokker–Planck equation which is the benchmark case of Wasserstein gradient flows. Extending the modified JKO scheme to other PDEs may stimulate applications of the theory to non-Euclidean spaces.
(iv) The effect of the A3 condition. Regularity for optimal transport costs requires a novel, but initially perplexing, condition known as A3 which was introduced by Ma, Trudinger, and Wang [41]. This condition was subsequently found to have numerous interesting interpretations and consequences [55–58]. The authors wonder if under this condition the associated smoothness of the optimal transport maps would lead to quantitative convergence rates.

## Data Availability

The manuscript has no associated data.

## Appendix A Weak formulations of the Fokker–Planck equation

We’ve relied on the following lemmas concerning the weak form of the Fokker–Planck equation. The global result is taken from Zhang’s work [30] and the result on precompact subsets is a straight forward adaptation.

### Lemma 12

Let (*N*, *g*) be a smooth Riemannian manifold and $M \subset N$ be a precompact submanifold each satisfying the hypothesis of Theorem 1 (i). Let $\rho \in L^1_{\text {loc}}([0,\infty ) \times M)$. Assume for every $\zeta \in C^\infty (M )$ satisfying the Neumann condition $\langle \nabla \zeta (x), \textbf{n}(x)\rangle = 0$ on $\partial M,$ and $\eta \in C^\infty _c([0,+\infty ))$ there holds

$$\begin{aligned} 0= -\eta (0) \int _{M } \zeta (x)&\rho _0(x) \textrm{dVol}_{g}- \int _0^\infty \int _{M } \zeta (x)\partial _t\eta (t) \rho (t,x) \textrm{dVol}_{g}\, \textrm{d}t\\&-\int _0^\infty \int _{M }( \Delta \zeta (x) - \langle \nabla \psi , \nabla \zeta \rangle )\eta (t) \rho (t,x) \textrm{dVol}_{g}\, \textrm{d}t. \end{aligned}$$

Then $\rho$ is the unique smooth solution of the Fokker–Planck equation satisfying the initial condition $\rho (0,x) = \rho _0$ and the Neumann boundary condition.

Recall from Section 5.1 that $m(\rho )$ denotes the second moment of $\rho$ about some fixed $x_0 \in M$.

### Lemma 13

Assume (*M*, *g*) is a complete Riemannian manifold with Ricci curvature bounded below and $\psi \in C^\infty (M)$ satisfies $\Vert \nabla \psi \Vert \le C(1+\psi )$. Let $\rho \in L^1_{\text {loc}}([0,\infty ) \times M )$ satisfy $\sup _{t}m(\rho ) < \infty$ and $E(\rho ),D(\rho ) < \infty$ for each $t > 0$. Assume for every $\zeta \in C_c^\infty (M )$ and $\eta \in C^\infty _c([0,+\infty ))$ there holds

$$\begin{aligned} 0= -\eta (0) \int _{M } \zeta (x)&\rho _0(x) \textrm{dVol}_{g}- \int _0^\infty \int _{M } \zeta (x)\partial _t\eta (t) \rho (t,x) \textrm{dVol}_{g}\, \textrm{d}t\\&-\int _0^\infty \int _{M } (\Delta \zeta (x) -\langle \nabla \psi , \nabla \zeta \rangle )\eta (t) \rho (t,x) \textrm{dVol}_{g}\, \textrm{d}t. \end{aligned}$$

Then $\rho$ is the unique smooth solution of the Fokker–Planck equation with initial condition $\rho _0$ and finite drift and second moment.

Both lemmas are standard. See, for example, the definition of weak solution in [30, Definition 4.2] as well as the bootstrap argument and uniqueness result [30, Theorem 4.5] for the global case. It is the uniqueness result that requires the condition $\Vert \nabla \psi \Vert \le C(1+\psi )$. The precompact case follows by similar techniques. One may also consult Friedman’s book [59, Ch. 3, Theorem 7] for the parabolic regularity theory and Figalli and Glaudo’s book for more details in the Euclidean case [34, Theorem 3.3.4].

## Footnotes

1 We note, however, that $d^2(x, y)$ is generally not globally smooth, so our conditions do not cover completely the Riemannian case as in [30, 31]. On the other hand, the existence of a smooth cost that induces a given metric was proved in [38].

2 Thus, in this proof, $\rho _0:= \rho ^\tau _{k+1}$, and is not the initial condition.

3 Moreover, when we use these calculations in the case *M* is a complete manifold the time derivative will be bounded on the support of $\zeta$.

4 More generally, a Hessian manifold is a Riemannian manifold whose metric can be locally expressed in the form (52). See [9] for an in-depth study. Here, we only consider the case where (52) holds globally, as otherwise the Bregman divergence is not globally defined.

5 See [44] for standard results in convex analysis.

6 The set *N* is relatively open so can be identified with an open convex set of some Euclidean space.

7 This is the canonical parameter under which the normal distribution is an exponential family [15].

8 Theorem 1 implies that the corresponding JKO scheme converges to the Riemannian Fokker–Planck where the metric is induced by (54). This answers (negatively) the conjecture in [52, Section 1.1] that the gradient flow is nonlocal.

9 We note that the former condition is also used in [54].
